# Methylenetetrahydrofolate Reductase Gene Polymorphism and Risk of Type 2 Diabetes Mellitus

**DOI:** 10.1371/journal.pone.0074521

**Published:** 2013-09-04

**Authors:** Jian-Hong Zhong, A. Chapin Rodríguez, Na-Na Yang, Le-Qun Li

**Affiliations:** 1 Surgery Oncology Department, Tumor Hospital of Guangxi Medical University, Nanning, People’s Republic of China; 2 Scientific Training Department, Association for Promotion of Multimedia Education (UMNA), Zagreb, Croatia; 3 Nursing Department, Tumor Hospital of Guangxi Medical University, Nanning, People’s Republic of China; University of Northampton, United Kingdom

## Abstract

**Objective:**

This review aimed to comprehensively assess the literature examining a possible link between the rs1801133 polymorphism (677C→T) in the gene encoding the methylenetetrahydrofolate reductase (MTHFR) gene and risk of type 2 diabetes mellitus (DM).

**Research Design and Methods:**

Several research databases were systematically searched for studies examining the genotype at the rs1801133 polymorphism in healthy control individuals and individuals with type 2 DM. Genotype frequency data were examined across all studies and across subsets of studies according to ethnicity and presence of serious DM-related complications. Odds ratios (ORs) and 95% confidence intervals (CIs) were calculated.

**Results:**

A total of 4855 individuals with type 2 DM and 5242 healthy controls from 15 countries comprising Asian, Caucasian and African ethnicities were found to satisfy the inclusion criteria and included in the review. Genotype at the rs1801133 polymorphism was not consistently associated with either increased or reduced risk of type 2 DM; the OR across all studies was 0.91 (95%CI 0.82 to 1.00) for the C- vs. T-allele, 0.88 (0.75 to 1.03) for CC vs. CT+TT, 0.82 (0.71 to 0.95) for CC vs. TT, and 1.15 (1.03 to 1.29) for TT vs. CC+CT. Similar results were found when the meta-analysis was repeated separately for each ethnic subgroup, and for subgroups with or without serious DM-related complications.

**Conclusions:**

There does not appear to be compelling evidence of an association between the genotype at the rs1801133 polymorphism of the MTHFR gene and risk of type 2 DM.

## Introduction

Diabetes mellitus (DM) is a global health epidemic, affecting approximately 171 million people in 2000 and projected to affect more than 360 million in 2030 [1]. Approximately 90% of people with DM have type 2 disease (T2DM) [2]. In contrast to T1DM, which is genetically inherited, T2DM has a complex aetiology that appears to involve numerous environmental risk factors and potentially some genetic risk factors.

Predicting T2DM risk is important because the disease can severely affect quality of life. T2DM is associated with a broad array of cardiovascular diseases, including retinopathy, nephropathy, neuropathy, acute myocardial infarction, stroke and atherosclerosis. It is important to diagnose and manage T2DM as early as possible to ensure therapeutic efficacy and avoid more serious long-term complications.

The rising prevalence of T2DM and the importance of early detection and management has led many investigators to search for environmental and genetic risk factors for T2DM and T2DM-related complications. Elevated plasma levels of homocysteine, a condition known as hyperhomocysteinaemia (HHcy), have been linked with such T2DM features as endothelial dysfunction and arterial stiffness [3], insulin resistance [4], [5], prothrombotic inflammation and hypercoagulability [6], macroangiopathy [4], [7] and nephropathy [8], [9]. HHcy has also been associated with atherosclerosis [10], coronary heart disease [11] and death [12] among individuals with T2DM.

The enzyme methylenetetrahydrofolate reductase (MTHFR) methylates homocysteine to generate methionine [13], and its dysfunction can lead to HHcy. Therefore numerous studies have investigated whether reduced MTHFR activity is a risk factor for T2DM. The single nucleotide polymorphism (SNP) rs1801133 (677C→T) leads to an Ala222Val substitution in the N-terminal catalytic domain of the enzyme. This mutation reduces enzyme activity, such that the activity in individuals with CT and TT genotypes is approximately 65% and 35%, respectively, that of individuals with the wild-type CC genotype [14], [15], [16]. As a result, individuals with the TT genotype have significantly higher Hcy levels than do individuals with CT and CC genotypes [17]. More recent genome-wide association studies (GWAS) have confirmed the association between rs1801133 genotype and homocysteine levels in healthy populations [18], [19].

Numerous studies around the world have examined whether an association exists between the 677C→T SNP and risk of T2DM. These studies have arrived at different conclusions, with some suggesting a significant association and others no association. This discrepancy is doubtless due in part to the wide variation in genotype frequencies at the rs1801133 locus. The TT genotype, for example, is present in 9–13% of Brazilians [20], 15–17% of north Indians [21], 18–20% of Chinese [7], and 20–30% of Turkish [22]. This makes it particularly important to systematically assess the association between this polymorphism and risk of T2DM across a range of ethnicities.

Despite the divergent results among single-country studies and strong evidence that rs1801133 genotype depends on ethnicity, no systematic review has been undertaken to determine conclusively whether this MTHFR SNP is associated with risk of T2DM, and whether the association is universal or specific to particular ethnic groups. To address this question as comprehensively as possible, we carried out a systematic review of case-control studies in the medical literature.

## Methods

### Literature Search Strategy

The most recent on-line versions of the following research databases were searched in April 2013 without language restrictions: Chinese National Knowledge Infrastructure (CNKI), Cochrane Library (http://onlinelibrary.wiley.com/cochranelibrary/search), Directory of Open-Access Journals (www.doaj.org), Embase, Public Library of Science (www.plosmedicine.org), PubMed, SciELO (www.scielo.org), Scopus, and Web of Knowledge. The following search terms were used to identify studies: “methylenetetrahydrofolate reductase” *or* MTHFR, gene *or* polymorphism *or* variation *or* genotype *or* genetic *or* mutation, diabetes *or* mellitus *or* “diabetes mellitus”. We also searched the Catalog of Published Genome-Wide Association Studies (www.genome.gov/gwastudies) of the US National Human Genome Research Institute.

### Inclusion Criteria

We included in the systematic review full-length research studies that satisfied the following criteria: (a) they assessed the association between T2DM and the 677C→T polymorphism of the MTHFR gene; (b) they used a case-control design in which cases were T2DM patients and controls were healthy individuals; and (c) they provided sufficient published data for estimating an odds ratio (OR) with a 95% confidence interval (95%CI). Conference abstracts or other forms of summary publication were not included.

If studies included case groups of DM patients with serious DM-related complications or control groups other than healthy individuals, data for those additional groups were not extracted. Serious DM-related complications, which included cardiovascular disease, coronary heart disease, nephropathy and diabetic retinopathy, were defined as complications aside from the more frequent clinical manifestations of T2DM such as hyperlipidaemia, hypertension and obesity. In the case of multiple studies apparently based on the same case or control population, we included only the study with the largest number of participants.

### Data Extraction

Two authors (J-HZ, ACR) independently extracted the following data from included studies: first author’s family name, year of publication, numbers of cases and controls, presence of serious complications among cases, duration of T2DM at the time of the study, Hardy-Weinberg equilibrium (HWE) of controls, and rs1801133 genotype frequencies in cases and controls. Extracted data were compared and discrepancies resolved by discussion.

### Statistical Methods and Bias Testing

The unadjusted OR with 95%CI was used to assess the strength of the association between the 677C→T polymorphism of the MTHFR gene and T2DM risk based on the genotype frequencies in cases and controls. The meta-analysis examined the association of different genotypes at 677C→T MTHFR with T2DM risk by comparing the C allele with the T allele, comparing homozygous genotypes, and applying recessive and dominant genetic models.

All statistical tests for this meta-analysis were performed using RevMan 5.14 (Cochrane Collaboration) and Stata 11.0 (StataCorp, College Station, USA). Pooled ORs were calculated using fixed- or random-effect models, and the significance of those ORs was assessed using the Z-test. The threshold for significance in the Z-test was defined as P<0.05. We used a chi squared-based Q-test to assess heterogeneity among studies. In this test, P>0.10 was taken to suggest that effect sizes were larger than those expected by chance [23], [24], indicating the absence of statistical heterogeneity. In this case, a pooled OR was calculated for each study using the fixed-effect model. Otherwise, the random-effect model was used. HWE in the control group was assessed using the asymptotic test, with P<0.05 considered significant.

Publication bias was assessed by visual inspection of Begg’s funnel plots. Small-study bias was assessed by Harbord’s modified test [25].

### Subgroup and Sensitivity Analysis

To detect associations that might be masked in the overall sample, we performed subgroup analyses based on subsets of the included studies defined according to ethnicity (African, Asian, and Caucasian) and according to whether the studies included T2DM cases with serious DM-related complications, such as cardiovascular disease, coronary heart disease, nephropathy and diabetic retinopathy. For subgroup analysis based on the presence or absence of serious DM-related complications, we defined two subgroups of studies: one subgroup in which the authors explicitly stated that such complications were absent, and another subgroup in which the authors either reported the presence of such complications or did not report on the presence or absence of complications at all. We also performed subgroup analysis separately on studies in which the MTHFR alleles in the control group were in HWE and on studies in which they were not in HWE. To assess the reliability of the outcomes in the meta-analysis, a sensitivity analysis was performed by excluding one study at a time.

## Results

**Figure 1:**
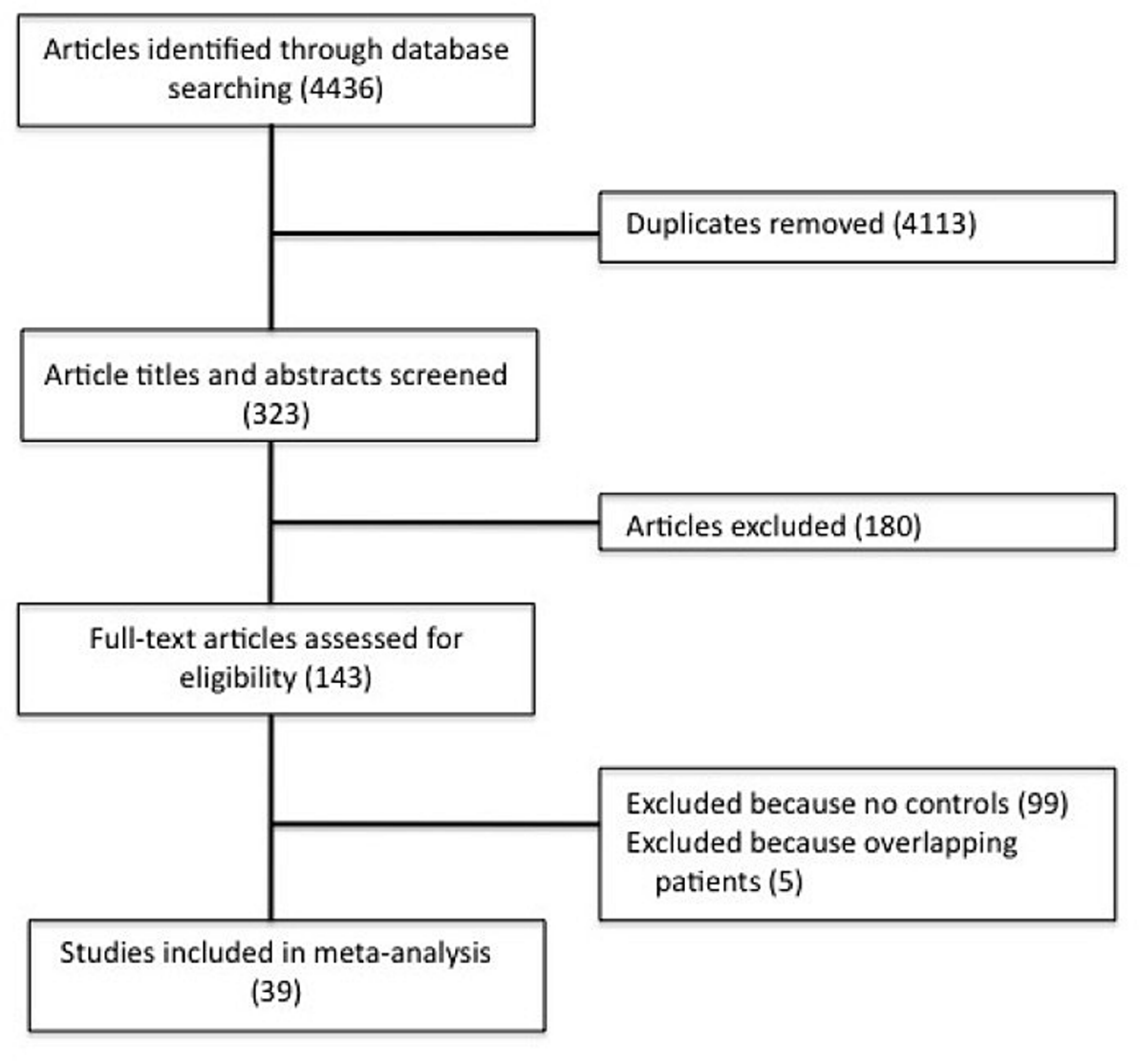
Flow chart of study selection.

Several research databases were searched without language restrictions to identify case-control studies assessing the possible association between the rs1801133 polymorphism in the MTHFR gene and risk of T2DM. A total of 4436 studies were identified, none of which was a GWAS. This list was reduced to 143 after removing duplicates and screening based on the title and abstract review. These articles were read in full, and 99 studies were removed because they did not include a healthy control group without T2DM, while another 5 studies were removed because they analysed overlapping patient populations. In the end, 39 studies were included in the meta-analysis (Fig. 1) [7], [8], [21], [22], [26]–[60]. The main characteristics of the included studies are shown in Table 1.

**Table 1:** Description of cases and controls and their genotypes at MTHFR polymorphism rs1801133.

Study (ref.)	Ethnicity	Cases/Controls	Serious DM-related complications^a^	Duration of DM (yr)^b^	Genotype by PCR-RFLP								P_HWE_ c
					Cases					Controls			
					CC	CT		TT		CC	CT	TT	
**Asian**													
Bazzaz 2010 [46]	Iranian	281/207	NR	NR	148	102		31		113	80	14	1.000
Chang 2011 [27]	Taiwanese	56/62	NR	NR	1	25		30		3	23	36	1.000
Chauhan 2012 [43]	Indian	1018/1006	NR	NR	31	310		677		45	335	626	1.000
Chen 2010 [29]	Chinese	40/55	No metabolic syndrome, inflammatory disease, kidney disease, or coronary heart disease	NR	23	13		4		34	17	4	0.439
Dai 2012 [26]	Chinese	60/60	No diabetic nephropathy	11.7±0.9	29	28		3		31	27	2	0.310
Eroglu 2007 [47]	Turkish	56/128	No diabetic nephropathy	NR	25	25		6		63	58	7	0.272
Hasegawa 2003 [45]	Japanese	62/200	No renal failure or macroangiopathy. Retinopathy present in 77.4% of cases.	18.5 (10–40)	23	32		7		78	96	26	0.762
Ho 2005 [36]	Taiwanese	20/42	Normal renal function; no deep vein thrombosis or coronary artery disease	NR	13	5		2		28	11	3	0.326
Luo 2009 [31]	Chinese	71/85	No cardiovascular disease or coronary heart disease	NR	39	26		6		43	31	11	0.204
Mao 2004 [37]	Chinese	41/47	No cerebrovascular disease	5.68±4.22	19	17		5		26	18	3	1.000
Mei 2012 [28]	Chinese	116/124	NR	NR	19	70		27		14	73	37	0.025
Movva 2011 [44]	Indian	100/100	No history or family history of renal complications	12.16±4.07	68	32		0		91	9	0	1.000
Raza 2012 [21]	Indian	87/88	NR	NR	35	37		15		49	26	13	0.009
Shi 2006 [33]	Chinese	104/110	No microvascular complications	NR	70	29		5		68	34	8	0.273
Sun 2006 [7]	Chinese	104/114	No overt nephropathy, cerebrovascular disease, coronary heart disease or peripheral vascular disease	7.04±4.71	60	27		17		63	31	20	<0.001
Tutuncu 2005 [48]	Turkish	87/91	Coronary artery disease present in 18% of cases; cerebrovascular disease, 3%; diabetic retinopathy, 18%; neuropathy, 9%.	8.7±7.1	39	37		11		47	39	5	0.593
Xiao 2006 [34]	Chinese	41/73	No nephropathy, cerebrovascular disease, coronary heart disease or peripheral vascular disease	NR	8	31		2		47	25	1	0.439
Xu 2003 [34]	Chinese	54/52	No diabetic nephropathy	6.77±4.08	24	21		9		20	25	7	1.000
Wang 2001 [42]	Chinese	117/85	No overt nephropathy	5.42±3.81	57	48		12		37	38	10	1.000
Wen 2008 [32]	Chinese	59/57	No nephropathy	4.8±5.3	21	32		6		27	25	5	1.000
Yang 2001 [41]	Chinese	102/62	No diabetic nephropathy, diabetic retinopathy, and other microvascular complications	>10	32	56		14		26	28	8	1.000
Yilmaz 2004 [22]	Turkish	249/214	Left ventricular hypertrophy present in 11.2% of cases	9.5±7.7	121	98		30		101	93	20	1.000
Yue 2006 [35]	Chinese	140/30	No diabetic nephropathy, diabetic retinopathy, or other microvascular complications	NR	43	76		21		17	11	2	1.000
Zhang 2002 [40]	Chinese	100/100	No macroangiopathy complications	NR	32	55		13		40	49	11	0.662
Zhang 2010 [30]	Chinese	206/194	NR	NR	73	98		35		53	103	38	0.388
Zhou 2004 [38]	Chinese	67/69	No macroangiopathy complications	NR	5	42		20		8	31	30	1.000
**Caucasian**													
Beneš 2001 [53]	Czech	49/209	No coronary artery disease	NR	24	20		5		86	106	17	0.062
Blüthner 1999 [55]	German and Polish	146/150	No diabetic nephropathy	17.0±7.0	63	65		18		67	68	15	0.853
Cenerelli 2002 [52]	Italy	30/43	No diabetic retinopathy, neuropathy, nephropathy, cardiovascular disease or coronary heart disease	3.1±6.5	8	14		8		13	21	9	1.000
Helfenstein 2005 [57]	Brazilian	50/56	No atherosclerosis, myocardial infarction, or diabetic retinopathy	NR	26	20		4		26	24	6	1.000
Książek 2004 [50]	Polish	155/170	No diabetic nephropathy. Retinopathy present in 8.4% of cases.	9.8±5.1	82	58		15		71	83	16	0.304
Mazza 2005 [54]	Italian	105/120	No coronary artery disease, cerebrovascular disease, kidney dysfunction, or diabetic proliferative retinopathy	11.4±8.0	35	47		23		35	66	19	0.264
Russo 2008 [49]	Italian	90/91	No cardiovascular disease	NR	28	42		20		23	50	18	0.403
Soares 2008 [56]	Brazilian	7/16	No coronary disease, stroke, or chronic renal failure	>1	5	2		0		9	5	2	0.530
Wirta 2002 [51]	Finnish	81/114	Background retinopathy present in 3.6% of cases	3 mos. (range, 1–11 mos.)	44	29		8		60	47	7	0.811
African													
Benrahma 2012 [58]	Moroccan	282/262	Neuropathic complications present in 30.5% of cases; cardiovascular complications, 17.4%; nephropathic complications, 2.5%.	NR	160		97		25	114	122	26	0.487
Mackawy 2011 [59]	Egyptian	40/40	No history of ischemic stroke	NR	24		10		6	32	6	2	0.090
Mehri 2010 [60]	Tunisian	115/116	History of cardiovascular disease present in 8.7% of cases; nephropathy, 26.1%; retinopathy, 26.1%; neuropathy, 19.1%.	9.3±5.7	50		49		16	66	38	12	0.095
Mtiraoui 2007 [8]	Tunisian	267/400	No nephropathy. Neuropathy present in 23.6% of cases; retinopathy, 19.1%.	12.3±4.1	152		79		36	270	94	36	<0.001

*Abbreviations:* NR, not reported; T2DM, type 2 diabetes mellitus; PCR-RFLP, polymerase chain reaction assay based on restriction fragment length polymorphism.

a Defined as serious complications aside from the more frequent clinical manifestations of T2DM such as hyperlipidaemia, hypertension and obesity.

b Reported as either “median (range)” or as “mean ± standard deviation”, unless indicated otherwise.

c P value for Hardy-Weinberg equilibrium for rs1801133 genotype among controls.

**Figure 2:**
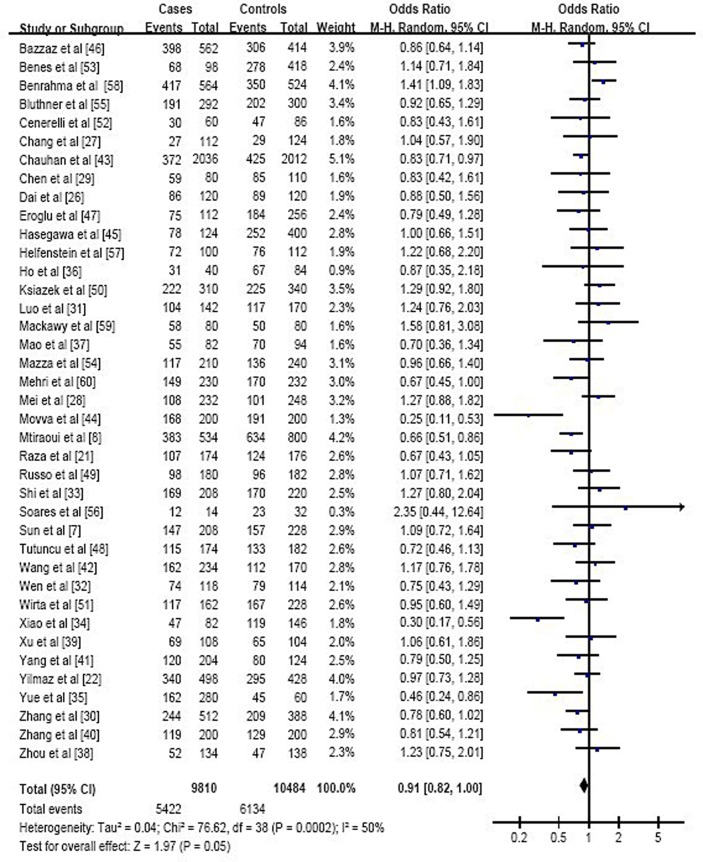
Forest plot assessing the potential association between the genotype at MTHFR polymorphism rs1801133 and risk of T2DM in all included studies (C-allele vs. T-allele).

These studies involved 4855 individuals with T2DM and 5242 healthy controls from 15 countries in Asia (26 studies), Europe (7), North Africa (4), and Brazil (2). Of the 39 studies, 14 were published in Chinese and 25 in English. Meta-analysis of all included studies indicated that the genotype at MTHFR polymorphism rs1801133 was not consistently associated with either increased or reduced risk of T2DM across the genetic models tested: the OR across all studies was 0.91 (95%CI 0.82 to 1.00) for the C- vs. T-allele (Fig. 2), 0.88 (0.75 to 1.03) for CC vs. CT+TT, 0.82 (0.71 to 0.95) for CC vs. TT, and 1.15 (1.03 to 1.29) for TT vs. CC+CT (Table 2).

**Table 2:** Overall and stratified meta-analyses of the association between methylenetetrahydrofolate reductase polymorphism 677C→T and risk of type 2 diabetes mellitus.

Genotype comparison	OR [95% CI]	Z (P value)	Heterogeneity of study design			Analysis model
			χ^2^	df (P value)	I^2^	
**All studies (4855 cases, 5242 controls)**						
C-allele vs. T-allele	0.91 [0.82, 1.00]	1.97 (0.05)	76.62	38 (<0.001)	50%	Random
CC vs. CT+TT	0.88 [0.75, 1.03]	1.56 (0.12)	95.21	38 (<0.001)	60%	Random
CC vs. TT	0.82 [0.71, 0.95]	2.62 (0.009)	35.40	37 (0.54)	0%	Fixed
TT vs. CC+CT	1.15 [1.03, 1.29]	2.57 (0.01)	26.07	37 (0.91)	0%	Fixed
**Subgroups by ethnicity**						
***Asian (3438 cases, 3455 controls)***						
C-allele vs. T-allele	0.86 [0.76, 0.96]	2.62 (0.009)	45.38	25 (0.008)	45%	Random
CC vs. CT+TT	0.81 [0.66, 0.98]	2.14 (0.03)	59.41	25 (<0.001)	58%	Random
CC vs. TT	0.82 [0.68, 0.99]	2.11 (0.04)	24.34	24 (0.44)	1%	Fixed
TT vs. CC+CT	1.12 [0.99, 1.27]	1.76 (0.08)	19.40	24 (0.73)	0%	Fixed
***Caucasian (713 cases, 969 controls)***						
C-allele vs. T-allele	1.06 [0.91, 1.22]	0.73 (0.47)	4.19	8 (0.84)	0%	Fixed
CC vs. CT+TT	1.22 [0.99, 1.49]	1.91 (0.06)	3.63	8 (0.89)	0%	Fixed
CC vs. TT	0.92 [0.67, 1.27]	0.51 (0.61)	2.25	8 (0.97)	0%	Fixed
TT vs. CC+CT	1.23 [0.91, 1.65]	1.35 (0.18)	2.06	8 (0.98)	0%	Fixed
***African (704 cases, 818 controls)***						
C-allele vs. T-allele	0.75 [0.46, 1.24]	1.12 (0.26)	23.04	3 (<0.001)	87%	Random
CC vs. CT+TT	0.75 [0.39, 1.43]	0.88 (0.38)	23.67	3 (<0.001)	87%	Random
CC vs. TT	0.70 [0.37, 1.31]	1.13 (0.26)	8.05	3 (0.05)	63%	Random
TT vs. CC+CT	1.32 [0.95, 1.83]	1.65 (0.10)	3.59	3 (0.31)	16%	Fixed
**Subgroups by DM-related complications**						
***T2DM with serious complications (3062 cases, 3248 controls)***						
C-allele vs. T-allele	0.91 [0.79, 1.04]	1.37 (0.17)	32.40	13 (0.002)	60%	Random
CC vs. CT+TT	0.96 [0.77, 1.20]	0.38 (0.71)	38.46	13 (<0.001)	66%	Random
CC vs. TT	0.83 [0.69, 1.00]	1.99 (0.05)	20.32	13 (0.09)	36%	Fixed
TT vs. CC+CT	1.17 [1.02, 1.33]	2.32 (0.02)	12.21	13 (0.51)	0%	Fixed
***T2DM without serious complications (1793 cases, 1994 controls)***						
C-allele vs. T-allele	0.90 [0.78, 1.04]	1.43 (0.15)	44.11	24 (0.007)	46%	Random
CC vs. CT+TT	0.83 [0.66, 1.03]	1.68 (0.09)	55.21	24 (<0.001)	57%	Random
CC vs. TT	0.82 [0.65, 1.03]	1.71 (0.09)	15.09	23 (0.89)	0%	Fixed
TT vs. CC+CT	1.13 [0.92, 1.39]	1.13 (0.26)	13.78	23 (0.93)	0%	Fixed
**Subgroups by HWE**						
***Alleles in control group in HWE (4281 cases, 4516 controls)***						
C-allele vs. T-allele	0.91 [0.82, 1.01]	1.75 (0.08)	64.89	34 (0.001)	48%	Random
CC vs. CT+TT	0.89 [0.75, 1.05]	1.36 (0.17)	83.85	34 (<0.001)	59%	Random
CC vs. TT	0.83 [0.70, 0.97]	2.33 (0.02)	28.57	33 (0.69)	0%	Fixed
TT vs. CC+CT	1.16 [1.04, 1.31]	2.53 (0.01)	21.44	33 (0.94)	0%	Fixed
***Alleles in control group not in HWE (574 cases, 726 controls)***						
C-allele vs. T-allele	0.88 [0.63, 1.24]	0.74 (0.46)	10.80	3 (0.01)	72%	Random
CC vs. CT+TT	0.83 [0.54, 1.26]	0.88 (0.38)	7.81	3 (0.05)	62%	Random
CC vs. TT	0.88 [0.51, 1.51]	0.46 (0.64)	6.80	3 (0.08)	56%	Random
TT vs. CC+CT	1.09 [0.81, 1.48]	0.57 (0.57)	4.52	3 (0.21)	34%	Fixed

To test the robustness of these findings, we recalculated ORs and 95% CIs across all studies after systematically removing each of them, one at a time. The results after deleting each study were similar to those obtained across all studies.

### Subgroup Analysis by Ethnicity

We performed subgroup analysis based on ethnicity in order to uncover any evidence of an association between the MTHFR SNP and risk of T2DM that might go undetected in the overall sample. We loosely classified the study populations as African, Asian, or Caucasian based on the majority ethnicity of the participants. Meta-analysis of each of the three subgroups failed to provide clear, consistent evidence that the genotype at MTHFR polymorphism rs1801133 was associated with either increased or reduced risk of T2DM (Table 2).

### Subgroup Analysis by Presence or Absence of Serious Complications of T2DM

Our original intention was to include only studies in which cases were T2DM without serious complications, which we defined as complications such as nephropathy, retinopathy, and coronary heart disease–in other words, complications aside from the hyperlipidaemia, HHcy, and obesity typically observed in patients with T2DM. Many studies, however, included cases with T2DM and serious complications, even when the study purported to assess only the association between the MTHFR SNP and T2DM per se. Therefore we divided the 39 studies into two subgroups: 14 studies that explicitly reported the presence of serious complications among cases or failed to report on such complications at all [8], [21], [22], [27], [28],[30],[43],[45],[46],[48],[50],[51],[58],[60], and 25 studies that explicitly reported the absence of serious complications [7], [26], [29], [31]–[42], [44], [47], [49], [52]–[57], [59]. Meta-analysis of these two subgroups, like the meta-analysis across all included studies, failed to provide clear, consistent evidence that the genotype at MTHFR polymorphism rs1801133 was associated with either increased or reduced risk of T2DM (Table 2).

Since the rs1801133 alleles in the control groups of several studies were not in HWE, we analysed those studies separately from those in which the alleles in the control groups were in HWE. The results were similar to those obtained across all included studies (Table 2).

### Bias Testing

**Figure 3:**
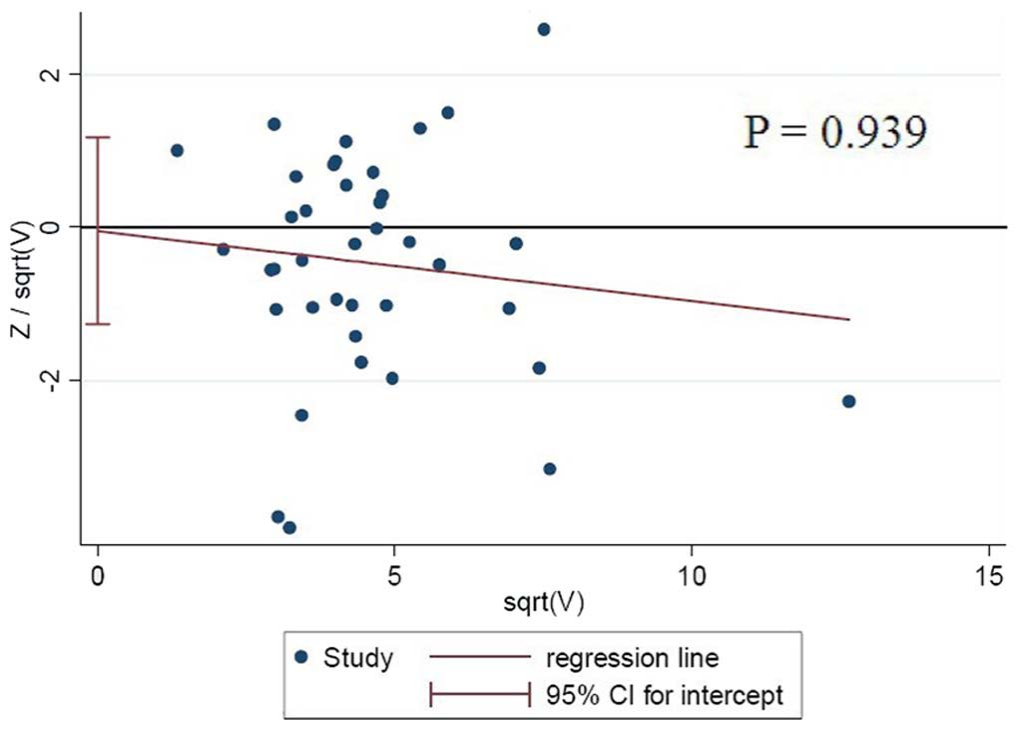
Analysis to detect small-scale study bias across all included studies, based on the allele contrast genetic model.

Begg’s funnel plots were prepared for the 39 studies to assess publication bias for studies about 677C→T MTHFR and T2DM risk. The shape of the funnel plots appeared to be symmetrical for allele contrast, homozygous comparison, and recessive and dominant genetic models, suggesting the absence of publication bias. Small-study bias tests showed no significant bias (P = 0.939, Figure 3).

## Discussion

This systematic review sought to assess the evidence for an association between the rs1801133 polymorphism in the MTHFR gene and risk of T2DM. We failed to find any clear, consistent evidence of such an association across all 39 studies conducted in 15 countries. In addition, no compelling evidence of an association was found specifically for African, Asian, or Caucasian populations, or specifically for populations with T2DM in the presence or absence of serious DM-related complications.

One of the challenges in conducting this systematic review was taking into account the clinical profile of the T2DM cases in the included studies. Such characterisation is important, because diabetes is a syndrome that can have far-reaching effects on various organ systems. For example, individuals with T2DM have approximately 2-fold higher risk of cardiovascular events than do individuals without diabetes [61]. This may make it difficult to determine whether the rs1801133 polymorphism is associated with T2DM, a T2DM-related complication or both. In order to isolate as much as possible an association between the MTHFR polymorphism and onset of T2DM per se, we analysed results separately for studies in which cases were explicitly described as having or lacking serious DM-related complications. The results of this subgroup meta-analysis were similar to those of the meta-analysis across all studies, further supporting the lack of an association between the MTHFR 677C→T SNP and risk of T2DM.

Our finding of a lack of association between this SNP and risk of T2DM contrasts with studies suggesting that this polymorphism is associated with certain serious DM-related complications. A meta-analysis of 29 studies found the TT genotype to be associated with moderately elevated risk for diabetic nephropathy and retinopathy [62]. Individual studies have reported the T allele to be associated with diabetic nephropathy [50], [63] and diabetic macroangiopathy [7], [45], but not with T2DM per se. These findings, in light of our meta-analysis results, highlight the need for rigourous, large-scale prospective studies that separate genetic risk of disease onset from genetic risk of complications. This work is crucial for clarifying whether MTHFR can affect long-term T2DM progression and patient prognosis.

Genotype frequencies at the rs1801133 locus of MTHFR vary widely by ethnicity [7], [20]–[22], raising the possibility that any association between this SNP and risk of T2DM may likewise depend on ethnicity. Therefore we repeated our meta-analysis separately for the ethnic groups that emerged from our literature searches: African, Asian, and Caucasian. We were restricted to these large, loosely defined ethnic categories because of the lack of detailed ethnicity data within the included studies. Meta-analysis results for each of the three ethnic groups failed to provide compelling evidence of an association between the MTHFR SNP and risk of T2DM. These findings are important because diabetes prevalence is projected to increase at substantially different rates in different ethnic groups. For example, diabetes prevalence is projected to increase between 2000 and 2030 by 26% in Italy, 71% in USA, 104% in China, 148% in Brazil, and 205% in Iran [1]. If this differential increase has a genetic basis, it seems unlikely to involve polymorphism in the MTHFR gene.

Another SNP in the MTHFR gene, 1298A→C (Glu429Ala), was reported to reduce enzyme activity based on studies of endogenous enzyme in lymphocyte extracts [15], [16], raising the possibility that it might be a risk factor for HHcy just like the 677C→T SNP. However, the 1298A→C SNP lowers MTHFR activity substantially less than does 677C→T [15], [16], [64], and biochemical studies with purified recombinant enzyme suggest that the originally reported lower activity for Glu429Ala enzyme may have been an artifact [65]. Furthermore, compelling evidence that the 1298A→C SNP is associated with HHcy is lacking [66]. The much milder effects observed with 1298A→C than with 677C→T are consistent with the fact that 1298A→C causes a mutation in the C-terminal regulatory domain of the enzyme, whereas 677C→T causes a mutation in the catalytic domain. Thus the 1298A→C SNP has not been the focus of studies of genetic risk factors of T2DM, and it was not considered in this systematic review.

The lack of an association between the 677C→T SNP and risk of T2DM may be consistent with studies calling into question whether HHcy plays a role in the disease. For example, although some authors have associated HHcy with macroangiopathy [4], [67], other authors have reported no such association [68]. Some studies have reported a positive association between total homocysteine levels and insulin levels in blood [69], while others have found a negative association [70] or no association at all [4]. To make the situation more complex, the negative effects of HHcy, at least with respect to increased risk of stroke, appear to be exacerbated by low folate [71]. Most of the studies included in the present systematic review did not analyze Hcy levels in cases and controls, making it difficult to gain a comprehensive picture. Of the studies that did examine whether Hcy levels were associated with the rs1801133 polymorphism, 11 found the TT genotype to be associated with higher Hcy levels than the CT or CC genotypes in patients with T2DM [7], [8], [31], [32], [35], [45], [46], [51], [54], [59], [60], while 6 found no such association [27], [48], [49], [52], [56], [57]. A meta-analysis published in 1998 concluded there was an association between the TT genotype and elevated plasma homocysteine levels in individuals with T2DM [17], while a more recent prospective study found no such association [72]. Thus, we second the conclusions of previous authors that the link between Hcy levels and T2DM remains unclear [48], [53], [56], [60]. The recommendation of the College of American Pathologists to measure Hcy only in patients with documented atherosclerotic disease [73] seems reasonable, given that HHcy is commonly accepted to be an independent risk factor for atherosclerosis and thromboembolism [74].

The findings in this systematic review are limited by the designs of the included studies. Only three studies [21], [43], [57] reported statistical power (76–98%), raising the possibility that other included studies were underpowered, which might lead to inaccuracy in our meta-analysis results. Nearly all included studies examined polymorphism only in the MTHFR gene, even though evidence suggests that this gene may interact with others in conferring risk or protection from diabetes. For example, the ID polymorphism in the gene encoding angiotensin-converting enzyme may act synergistically with the MTHFR 677C→T polymorphism to enhance diabetes risk [60]. Another design limitation is that most included studies looked only at a *genetic* association between MTHFR SNP genotype and T2DM risk. This is different from examining the downstream effects of different MTHFR SNP genotypes. For example, since more than half the included studies did not examine plasma Hcy levels in individuals with different genotypes, it is difficult to gain further insight into whether these levels are directly linked to risk of T2DM and thereby resolve the contradictory information in the literature. Several studies in our meta-analysis included patients with T2DM who had been managing their disease with medication, while other studies excluded such patients; studies also diverged significantly with respect to how long their participants had been living with the disease. Further studies should aim to control for as many possible confounders as possible.

Our findings fail to provide compelling evidence for an association between the MTHFR polymorphism rs1801133 and risk of T2DM, regardless of the ethnicity of the patient or the presence of serious DM-related complications. The possibility remains that MTHFR does affect risk of T2DM in concert with other genetic risk factors, which might include the 1298A→C SNP or additional polymorphisms that recent GWAS have linked to homocysteine levels [18].

Reporting of this meta-analysis has been guided by the PRISMA statement (Checklist S1).

## Supporting Information

Checklist S1 PRISMA 2009 Checklist.
(DOC)
